# Preparation of highly efficient p-doped porous camellia shell-based activated carbon and its adsorption of carotenoids in camellia oil

**DOI:** 10.3389/fnut.2022.1058025

**Published:** 2022-11-16

**Authors:** Run Tian, Yang Liu, Danyu Cao, Lili Gai, Nan Du, Jiangyu Yin, Dongbin Hu, Haiqin Lu, Wen Li, Kai Li

**Affiliations:** ^1^College of Light Industry and Food Engineering, Guangxi University, Nanning, China; ^2^Guangxi Key Laboratory of Chemistry and Engineering of Forest Products, School of Chemistry and Chemical Engineering, Guangxi University for Nationalities, Nanning, China; ^3^Provincial and Ministerial Collaborative Innovation Center for Sugar Industry, Nanning, China

**Keywords:** camellia oil, adsorption, activated carbon, interaction, mechanism

## Abstract

The vegetable oil industry is limited by the high cost of the refining process, and the camellia shells (CS) are beneficial to the development of the industry as a biomass raw material for camellia oil decolorization. In this study, CS-based p-doped porous activated carbon (CSHAC) obtained after the pyrolysis of H_3_PO_4_-laden CS-hydrochar (CSH) was used for the adsorption of carotenoids in camellia oil. The results showed that the adsorption efficiency of CSHAC for carotenoids was 96.5% compared to 67–87% for commercial decolorizers, and exhibited a fast adsorption rate (20 min). The results of adsorption isotherms indicated that the adsorption of carotenoids on CSHAC occurred through a multi-layer process. Furthermore, the analysis of adsorption kinetics showed that the adsorption of carotenoids by CSHAC was a complex process involving physical and chemical reactions, and chemisorption was the dominant kinetic mechanism. This superior performance of CSHAC in adsorbing carotenoids was attributed to its micro-mesoporous structure, hydrophobicity, and numerous active sites.

## Introduction

Carotenoids are derivatives of 40-carbon isoprene, which can be derived from a variety of classes due to different substituents. Common carotenoids in vegetable oils are β-carotene and lutein, which appear yellow, orange, or red (1). Although carotenoids have health-benefiting antioxidant properties, they show pro-oxidant activity at high temperatures during vegetable oil refining process (2). Therefore, research on the removal of carotenoids is necessary for the subsequent processing of refined vegetable oils, to meet oil quality standards and to promote a healthy diet.

Activated clay and activated carbon have been utilized in oil decolorization (3). However, in the refining process, acid-activated activated clay catalyzes the oxidation of oil in oxygenated and high-temperature environment (4). In contrast, carbon materials are considered to be a better choice. However, the existing commercial activated carbon (CAC) is not satisfactory for the decolorization efficiency of vegetable oils, and the production of high-efficiency carbon materials requires expensive precursors and equipment. Biomass has attracted extensive research attention due to its distinctive advantages, including resource-rich, clean, and eco-friendly (5). Bamboos (6), peanut shells (7), rubber seed shells (8), and pinewoods (9) have been used to produce effective activated carbon, which is used as an adsorbent in many applications and can eliminate the waste of certain resources. However, detailed studies on the adsorption of carotenoids from vegetable oils by activated carbon are rare.

Hydrothermal carbonisation (HTC) refers to operation in a sealed reactor under mild subcritical reaction conditions below 250°C to promote the dissolution of lignocellulosic components into water (10). The HTC process produces hydrochar with a high aromatic structure and a high content of oxygen-containing functional groups (11). A study revealed that carotenoids were oxidized upon adsorption (12). Therefore, this process is capable of facilitating the removal of pigments. Increasing the micro-mesoporous structure, surface area, and active groups of carbon materials can also improve their adsorption efficiency (13). Bentonite clays with predominantly mesoporous structural characteristics have been fabricated, and the researchers believed that the average pore size of clay particles affected their oil-bleaching capacity (14). Another study investigated the bleaching capacity of activated animal bone with a surface area of 593.27 m^2^/g on palm oil, but its efficiency at 120°C was only 75.14% (15). Some researchers studied the thermo-mechanical process of bleaching oil on adsorbents constituted of 5% activated carbon and 95% activated earth (16). However, the above studies of composite adsorbents did not fully demonstrate the mechanism of adsorption between all carbon-based adsorbents and pigments.

Camellia shells (CS) can be used as decolorizers for refined camellia oil. These shells have great potential for mass production, and comprehensive utilization of agricultural by-products can reduce industrial costs, which is conducive to increasing their economic added value. Moreover, considering the rich mesoporous structure of the shell, it is a promising material for the production of porous carbon. There is minimal research on the HTC method for yielding high-efficient activated carbon from CS as vegetable oil decolorizing agents. Although the effectiveness of single-step carbonisation is acceptable, HTC-assisted carbonisation has been investigated to optimize consumption of energy, as well as to biomass applications, since biomass has a high percentage of water. Therefore, pre-treatment with the HTC process before pyrolysis is expected to produce carbon materials with improved physicochemical performances.

In this study, p-doped porous activated carbon (CSHAC) with high specific surface area and ample surface groups was prepared from CS. The adsorption mechanisms of adsorbents for carotenoids of camellia oil were analyzed through adsorption kinetics, isotherms, and thermodynamics. This is the first report on CS as a precursor for the synthesis of p-doped porous carbon for removing carotenoids from vegetable oils, to the best of our knowledge. This work introduces a new type of biomass for rapid carotenoid adsorption. It can also provide a reference for decolorization mechanisms for vegetable oils.

## Materials and methods

### Chemicals and materials

Crude camellia oil was supplied by the Zhongzhou Company, China. Camellia shells were from Kaihua County, Quzhou City, Zhejiang Province, China. Camellia shells were pretreated by washing thoroughly with distilled water and drying in the sun for 8 h before being crushed and sieved with a 100 μm mesh in a stable arm crusher. Phosphoric acid (H_3_PO_4_, 85%) was purchased from Guangdong Guanghua Sci-Tech Co., Ltd. Commercial activated clay (CC) was purchased from Gongyi Runsen Water Treatment Materials Co., Ltd. Commercial activated carbon was purchased from Dongguan Hongsheng Activated Carbon Co., Ltd.

### Preparation of CSHAC

CSHAC was obtained using the HTC method, then acidified with 20% H_3_PO_4_ and carbonized (17). The HTC method was performed in an autoclave reactor. First, 5 g of CS and 50 ml of distilled water were mixed well and sealed in a stainless steel sample reactor. The reactor was then heated at 200°C for 1 day. Next, the solid product, namely CS-hydrochar (CSH), was flushed with distilled water and whereafter dried at 105°C for 1 day. The process was continued by stirring CSH with 50 ml of 20% H_3_PO_4_ at ambient temperature for 5 min and drying at 105°C for 1 day. The dried products were then carbonized at 600°C at a heating rate of 5°C min ^–1^ with a continuous N_2_ flow for 3 h. The collected product was designated CSHAC after being rinsed with distilled water to remove excess acid and by-products up to pH 7.

### Preparation of refined camellia oil

The refined camellia oil contained 35.2 mg/kg carotenoids, which was obtained by filtering the crude camellia oil through a ceramic membrane with a pore size of 0.14 μm to remove possible suspended impurities.

### Batch adsorption studies

Batch adsorption tests were performed to assess the performance of CSHAC for the adsorption of carotenoids. All the adsorption studies were performed in a temperature-controlled incubator shaker with consistent stirring (150 rpm) (ZQZY-85CN, Zhichu, China). A conical flask was used to place refined camellia oil during the experiments. The influences of important factors, for instance, sorbent material, sorbent dosage, temperature, and contact time on carotenoid adsorption were investigated. Each adsorption experiment was repeated thrice, and the results were averaged. After adsorption, the samples were split from the oil by transiting them through filter paper. Based on the British Standard Methods of Analysis (BS, 1993), the carotenoid concentrations of samples were determined at 454 nm with cyclohexane as the reference solution using a spectrophotometer (SP-752, Spectrum, China). The total carotenoid content was expressed as β-carotene equivalents. Later, 1 g of refined camellia oil was weighed, dissolved in cyclohexane, and diluted to a final volume of 100 ml. To acquire reliable results, the suspended impurities were filtered through the membrane (0.45 μm) and residual carotenoid concentrations were detected. The followings are the adsorption capacities of carotenoids on CSHAC at time *t*(*q*_*t*_) and equilibrium (*q*_*e*_) and the carotenoid removal rate (*R*):


(1) $$q_{t}=\frac{\left(C_{0}-C_{t}\right)\times V}{m}$$

(2) $$q_{e}=\frac{\left(C_{0}-C_{e}\right)\times V}{m}$$

(3) $$R=\frac{C_{0}-C_{t}}{C_{0}}\times 100\%$$

where *C*_0_ (mg/L), *C*_*t*_ (mg/L), and *C*_*e*_ (mg/L) show the concentrations of carotenoid at the original time, equilibrium, and time *t*, respectively; *m* (*g*) is the adsorbent dosage; *V* (L) is refined camellia oil volume.

### Analysis

The N_2_ adsorption-desorption analysis was determined by using the Brunauer–Emmett–Teller (BET; ASAP2460; Micromeritics, USA) equation. The surface features of samples were observed using scanning electron microscopy (SEM; Zeiss Gemini 300, Germany). The X-ray diffraction (XRD) patterns in the scanning range of 5–80° of the samples were measured using an X-ray diffractometer (SmartLab3KW, Rigaku, Japan). Raman spectroscopy was performed using a DORIBA Spectra instrument operating with a 532 nm green laser (Horiba LabRAM HR Evolution, Japan). The mapping images were provided with an X-ray spectrometer [energy-dispersive X-ray spectrometer (EDX); SMART, EDAX Inc., USA]. X-ray photoelectron spectrometry (XPS) was used to analyze compositions of sample surfaces (K-Alpha, Thermo Fisher Scientific, USA). Contact angles analysis of samples was obtained by using contact angle equipment (DSA100; KRUSS, Germany). Fourier transform infrared spectrometer (FTIR) was used to detect the functional groups of the samples (IRTracer-100, Shimadzu, Japan). An ultraviolet detector was used for identifying compounds at a wavelength of 454 nm. The free fatty acid contents of the samples were determined using the AOCS Official Method, C. (2017). 3d-63. Acid value fats and oils. The peroxide values of the samples were determined using the national standard (GB 5009.227–2016).

## Results and discussion

### Characterisation of CSHAC

#### Scanning electron microscopy-energy-dispersive X-ray spectrometer studies

**FIGURE 1:**
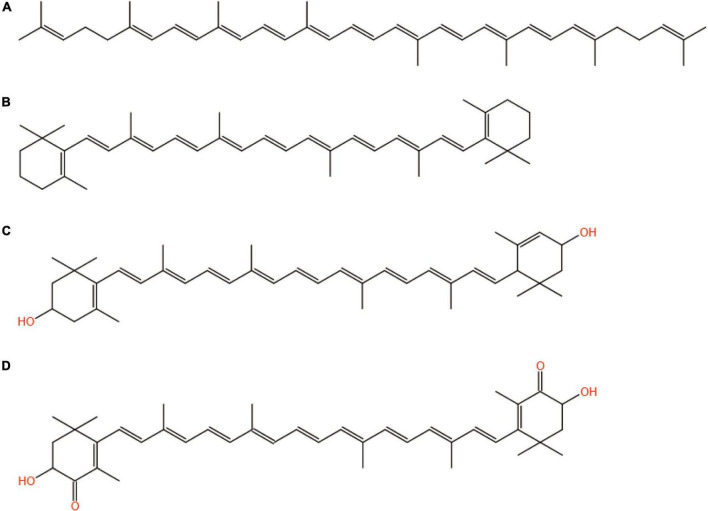
Structures of **(A)** lycopene, **(B)** β-carotene, **(C)** lutein, and **(D)** astaxanthin.

**FIGURE 2:**
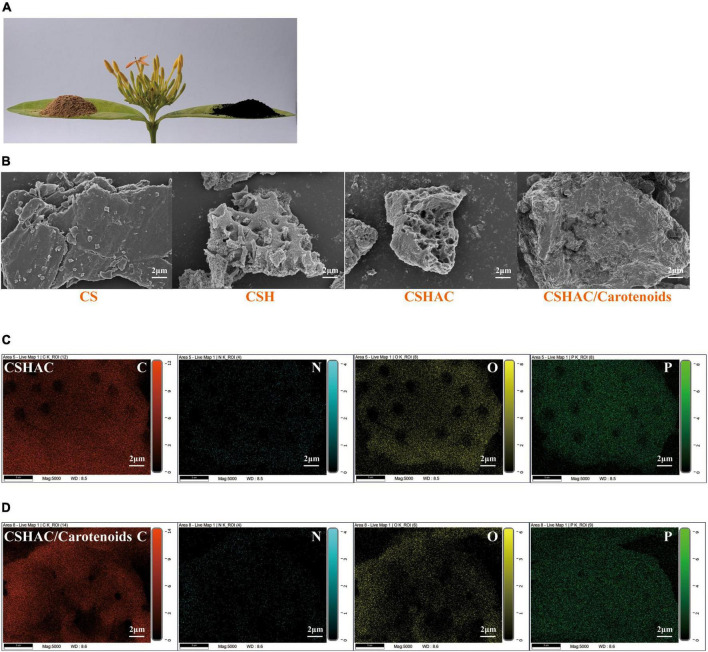
**(A)** Images of CS and CSHAC on *Ixora chinensis* leaves. **(B)** SEM images of CS, CSH, CSHAC, and CSHAC/carotenoids. EDX images of **(C)** CSHAC and **(D)** CSHAC/carotenoids.

The structures of the four common carotenoids were depicted in Figure 1. As depicted in Figure 2A, CS and CSHAC could be easily placed on the leaves of *Ixora chinensis*. Unnoteworthy deformation of the leaf manifested that the density of prepared CSHAC did not change significantly compared with CS. Surface morphologies of CS, CSH, CSHAC, and CSHAC/carotenoids were characterized using SEM. Figure 2B exhibited the typical SEM images. The pristine CS exhibited a relatively smooth surface. In comparison, the HTC process developed some pore space with a multilevel layered structure on the CSH surface. CSHAC exhibited an abundant network-like porous structure that could expose more available active sites for the uptake of carotenoids during the adsorption process. The lignin skeleton was exposed during acidification and pyrolysis (18). The pore structure of CSHAC was well developed, forming delamination within the pore channel, and even causing surface etching of CSHAC. As depicted in Figure 2B, the morphology of CSHAC changed upon carotenoid adsorption. At the end of the adsorption process, the surface of CSHAC was wrapped with the refined camellia oil pigments, and the pore structure was no longer clearly and visibly exposed.

Figures 2C,D exhibited the EDX element mapping images for CSHAC and CSHAC/carotenoids. The EDX spectra of CSHAC were mainly composed of C, O, N, and P. It was evident that the P element were successfully doped into the carbon skeleton after biomass pyrolysis and evenly distributed on the CSHAC surface, which ensured that CSHAC had a high chemical reactivity for carotenoid adsorption. Compared with CSHAC, the C, N, and O contents of CSHAC/carotenoids were significantly different, revealing that the chemical composition of CSHAC changed after the carotenoid adsorption process.

#### Brunauer–Emmett–Teller analysis

**FIGURE 3:**
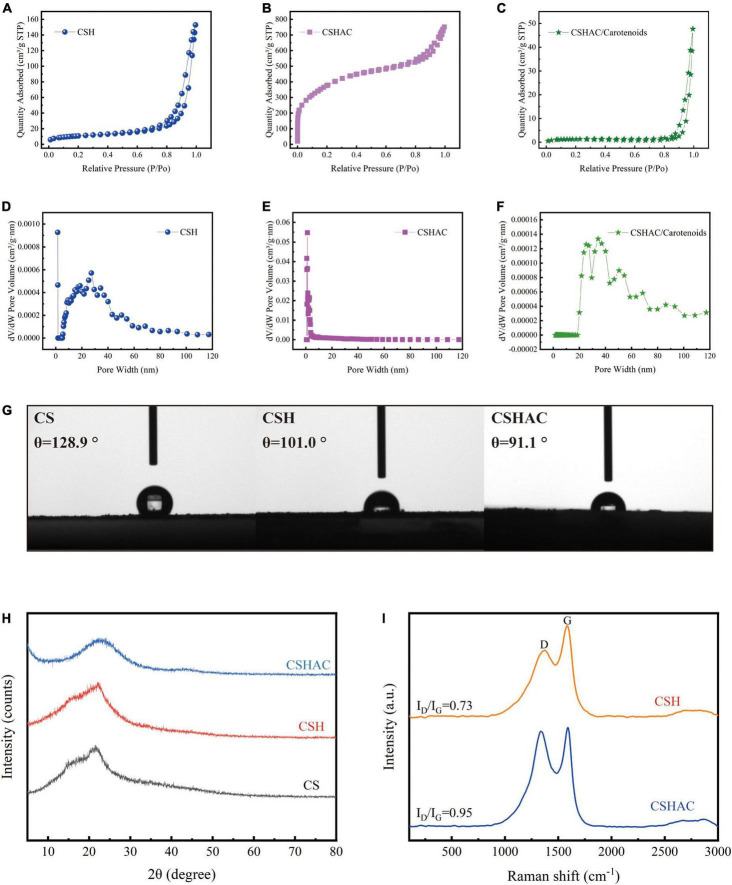
N_2_ adsorption-desorption isotherms for **(A)** CS, **(B)** CSH, and **(C)** CSHAC. The pore size distribution curves of **(D)** CSH, **(E)** CSHAC, and **(F)** CSHAC/carotenoids. **(G)** Contact angles determined for CS, CSH, and CSHAC. **(H)** X-ray diffraction patterns of CS, CSH, and CSHAC. **(I)** Raman spectra of CSH and CSHAC.

The results of the N_2_ adsorption-desorption isotherms investigation were exhibited in Figures 3A–F. It was evident in Figure 3A that the CSH adsorbent exhibited a typical type IV isotherm with an H_3_ hysteresis loop, proving that the CSH process transformed the adsorbent into a mesoporous material with inter-granular voids or slit pores (IUPAC classification). The adsorption isotherms for CSHAC (Figure 3B) exhibited a sharp rise in the low relative pressure range (*p*/*p*_0_ < 0.1) and represented a hysteresis loop at a relative pressure of 0.45, which indicated that the isotherms belonged to the combination of type I and type IV isotherms (19). Moreover, the hysteresis loop corresponded to the H_4_ loop. These results implied that the prepared CSHAC was micro-mesoporous carbons (20). The isotherms exhibited an obvious upturn at *p*/*p*_0_ higher than 0.95, indicating that the adsorbent contained macropores. These findings were consistent with the results of the SEM investigation and the reasons for the pore collapse of CSH and CSHAC proposed by the authors. As depicted in Figure 3C, the adsorption-desorption isotherm of CSHAC/carotenoids was a type IV isotherm of the H_3_ hysteresis loop, proving that carotenoid adsorption changed the pore structure of the adsorbent. This might be related to the physical or chemical adsorption of carotenoids. The specific surface areas, total pore volumes, and average pore sizes of the samples were calculated (see Table 1 for details). The specific surface area and pore volume of CSHAC were significantly higher than those of CSH, demonstrating that CSHAC was well activated and could facilitate the capture of colorants from refined camellia oil. By comparing the difference in average pore sizes between CSHAC and CSH, we speculated that the pore size distributions of adsorbent might affect adsorption efficiency (13). The specific surface area and pore volume of CSHAC after adsorption were significantly lower than before, and the microporous structure disappeared, indicating that the pigments diffused through the pore surface and inside the pores. This finding also indicated that CSHAC exhibited good pore-mediated adsorption.

**TABLE 1:** Physicochemical properties of CS, CSH, CSHAC, and CSHAC/carotenoids.

Adsorbent	Specific surface area (m^2^/g)	Pore volume (cm^3^/g)			Average pore size (nm)
		
		Vtot	Vmic	Vmes	
CS	0.19	0.012	–	–	261
CSH	37.87	0.263	0.005	0.231	31
CSHAC	1,359.25	1.162	0.451	0.922	4.1
CSHAC/ Carotenoids	3.86	0.074	0.002	0.073	76

Vtot, total pore volume; Vmic, micropore volume; Vmes, mesopore volume.

#### Contact angle measurement

As shown in Figure 3G, a contact angle measurement apparatus was used for studying the hydrophilicities/hydrophobicities of CS, CSH, and CSHAC. The contact angle of the CS surface was 128.9°, which manifested hydrophobicity. After the HTC, acidification, and pyrolysis processes, the contact angles of the CSH (101.0°) and CSHAC (91.1°) surfaces were slightly lower than those of the CS surface. The decrease in hydrophobicity could be ascribed to an increase in the porosity and number of functional groups of CSH and CSHAC (21). The adsorption process proceeded in two steps (22). First, the adsorbate was transferred through the liquid film around the adsorbent. Second, the adsorbate diffused through the pores of the adsorbent and then adsorption took place in the active sites of the sorbent. The hydrophobicity of sorbent behaved a significant part in the sorption process (23). Under hydrophobic conditions, the diffusion rates through the liquid film and pore could be accelerated to achieve adsorption equilibrium (section “Effect of contact time”). Hence, high carotenoid removal and rapid adsorptive rates could be accomplished during the disposal of colorants in refined camellia oil.

#### X-ray diffraction analysis

The crystallinities of CS, CSH, and CSHAC were examined using XRD spectroscopy in the range of 5–80°. The results were shown in Figure 3H. Remarkably, the XRD patterns of the samples after one- and two-step processing exhibited XRD patterns similar to those of the raw material, indicating that their natural structure was preserved. Two broad peaks centered at approximately 2θ = 23° and 43° corresponded to the diffraction peaks of the (002) and (100) planes of the amorphous carbon material (24). The absence of a sharp peak from the pattern further signified that the activated carbon had a lower degree of graphitisation; at such a temperature, the graphitic structure had not developed (25). Compared the XRD patterns of CSHAC with CSH, it was distinct that the diffraction at (002) was less broad, which meant a higher degree of graphitisation. This might be the result of a complete reaction between H_3_PO_4_ and CSH at a high carbonization temperature. These results indicated that the characteristics of the high specific surface area and microporous structure of the adsorbent were closely related to the orientation of the graphite layer and the expansion and distribution of the carbon lattice (26).

#### Raman analysis

Raman analysis of carbon exhibited a further comprehension of the structural defects on the surface of the materials. Figure 3I depicted the results of the Raman analysis of the CSH and CSHAC samples. The Raman spectra for CSH showed two well-defined peaks at 1,383 cm ^–1^ (D peak) and 1,588 cm^–1^ (G peak), respectively (27). The peak at around 2,500–3,200 cm^–1^ was due to the existence of either the –OH group of carboxylic acid or disordered substances (28). The D peak indicated disorders in the graphitic structure of carbon materials, while the G peak represented the graphitic structure of carbon materials caused by the vibration of sp^2^ hybrid carbon atoms in both rings and chains (29). The relative intensity ratio of the D peak to the G peak (ID/IG) was approximately 0.73. Furthermore, the Raman spectra of CSHAC exhibited an increased ID/IG ratio of 0.95, which obviously established the presence of more structural disorders in CSHAC. Acid treatment endowed CSHAC with more defective structures, and thus the increased number of active sites enhanced its adsorption capacity.

#### Fourier transform infrared spectrometer analysis

**FIGURE 4:**
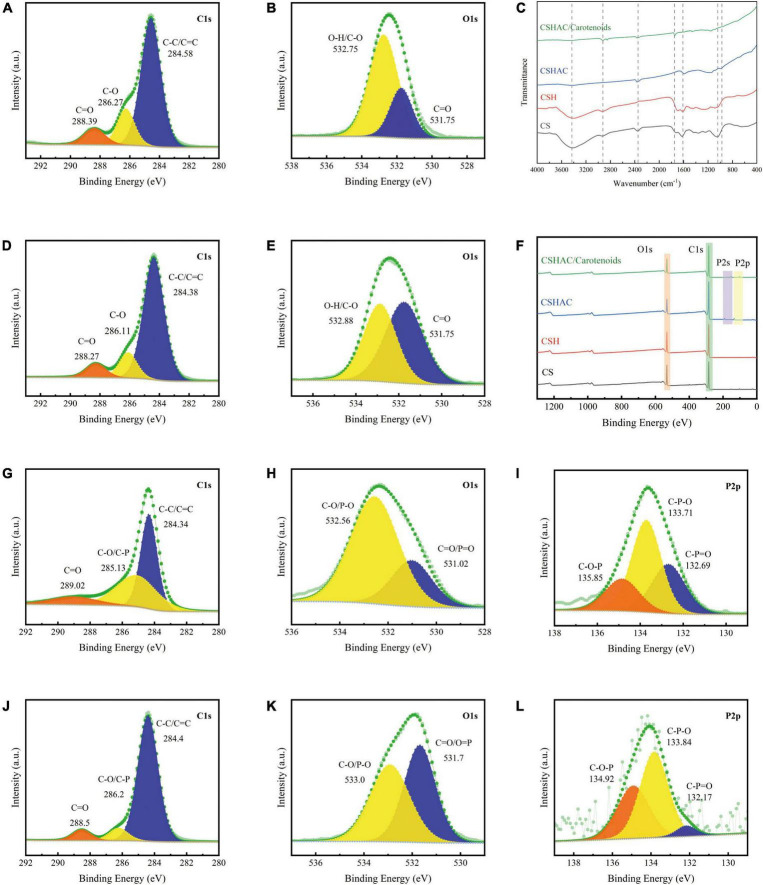
**(A)** C1s and **(B)** O1s spectra of CS. **(C)** FTIR spectra of CS, CSH, CSHAC, and CSHAC/carotenoids. **(D)** C1s and **(E)** O1s spectra of CSH. **(F)** XPS survey spectra of CS, CSH, CSHAC, and CSHAC/carotenoids. **(G)** C1s, **(H)** O1s, and **(I)** P2p spectra of CSHAC. **(J)** C1s, **(K)** O1s, and **(L)** P2p spectra of CSHAC/carotenoids.

The FTIR spectra of CS, CSH, CSHAC, and CSHAC/carotenoids were depicted in Figure 4C. As the processing steps increased, the intensity of the absorption peak at 3,417 cm^–1^ decreased, which implied a decrease in the content of –OH (30). After the activation of CS at 600°C, the inter- and intra-molecular dehydration reactions of cellulose and hemi-cellulose were intense, and the hydroxyl group was released in the form of water. The higher the temperature, the more the hydroxyl groups were released, and the lower the intensity of their absorption peak in the infrared spectrum. The oxygen content decreased from 20.16 to 17.58% with the activation of the phosphoric acid solution (Table 3). This result was attributed to the dehydration of phosphoric acid, which could eliminate O and H from carbon. The –CH_3_ and –CH_2_ stretching vibrations of aliphatic hydrocarbons or cycloalkanes were observed near 2,933 cm^–1^ (31). The intensity of this absorption peak in the CSHAC spectrum was not significant when compared with that of CS, mainly because the sample underwent a demethylation reaction after activation at 600°C, producing H_2_, CH_4_, and C_2_H_6_ gases (32). The peak was found at 1,625 cm^–1^ (C=C stretching), which represented the possible aromatic skeleton in CSHAC and indicated that CSHAC had some hydrophobicity (20). This result was similar to the contact angle results (section “X-ray diffraction analysis”). The positions of C=C (shifted from 1,625 to 1,734 cm^–1^) on CSHAC changed after carotenoid adsorption, which manifested that they might participate in carotenoid complexation. The 1,500–1,100 cm^–1^ bands were the absorption peaks of C-O-C, C-H, C-OH, and other stretching vibrations (33). The 1,039 cm^–1^ band corresponded to C-O stretching, and 977 cm^–1^ was the band between acid and carbon (33). This indicated that the P element doped into CSH might be combined with the C and O element. After the high-temperature treatment, some C-OH groups in the oil shell charcoal were broken, and the intensity of the absorption peaks was reduced. The results indicated that activation promoted the pyrolysis of –CH_3_ and –OH, however, the oxygen-containing functional groups in the carbon materials were retained. The unsaturated aromatic units on carotenoids and the oxygen-containing functional groups on CSHAC exhibited significant electron absorption capacity and could be considered as π-electron acceptors, and hydroxyl and methyl groups could act as π-electron donors. The π–π interactions enhance the adsorption capacity of carotenoids on CSHAC.

#### X-ray photoelectron spectrometry analysis

The surface compositions of the CS, CSH, CSHAC, and CSHAC/carotenoids were analyzed using XPS. The results were shown in Figure 4. The survey spectra of all samples exhibited pronounced peaks of carbon and oxygen (Figure 4F). There were two other weak peaks at approximately 191.0 and 132.75 eV that were observed in the survey curves of both CSHAC and CSHAC/carotenoids, which corresponded to the binding energies of P2s and P2p spectra. These came from the H_3_PO_4_ activation process (20). The C1s spectra of CS and CSH were divided into three contributions: C-C/C=C, C-O, and C=O, whose predominant chemical states were C-C/C=C (Figures 4A,D) (34). Their percentages were ascertained on the strength of the depth profiles acquired using XPS. When compared with CS, CSH had a higher C-C/C=C content, indicating higher aromaticity. The O1s peak recorded for CS and CSH were split into two peaks at 532.75 (C-O/O-H) and 531.75 eV (C=O), respectively (Figures 4B–E) (14). The result of the O1s spectrum profile recorded for CSH suggested that the proportion of each component of CSH was different from that observed for CS due to reactions such as dehydration and decarboxylation.

The C1s spectra were recorded for CSHAC (Figure 4G). The peaks corresponded to C=O (289.02 eV), C-O/C-P (285.13 eV), and C-C/C=C (284.34 eV) of CSH, respectively (35). The presence of the peak at 284.34 eV (C-C/C=C) indicated that the CSHAC surface had a certain degree of aromatic structure. Theoretically, phosphoric acid activation of CSH led to oxidation and catalytic dehydration reactions, as confirmed by the increased molar percentages of C-O and C=O in the C1s spectra of CSHAC (Figures 4D,G). The O1s spectrum of CSHAC was depicted in Figure 4H. The peaks of C-O/P-O (532.56 eV) and C=O/P=O (531.06 eV) were ascribed to the CSH after acidification and carbonisation, respectively. As shown in Figure 4I, the P2p spectrum of CSHAC could be parsed into C-O-P, C-P-O, and C-P=O components at 135.85, 133.71, and 132.65 eV, respectively (36).

The C1s spectrum recorded for CSHAC/carotenoids was depicted in Figure 4J. Compared with CSHAC, the C-C/C=C content of CSHAC/carotenoids increased after carotenoid adsorption owing to the carotenoids attached to the CSHAC surface. Consistent with the above FTIR analysis, π–π interactions promoted the adsorption of carotenoids. The result of the O1s spectrum recorded for CSHAC/carotenoids (Figure 4K) indicated that the ratio and binding energies of each component of CSHAC/carotenoids differed from those of CSHAC (Figure 4H). Evidently, the C-O content was reduced after carotenoid adsorption, which indicated that it participated in carotenoid complexation. Compared the P2p spectra of CSHAC (Figure 4I) with CSHAC/carotenoids (Figure 4L). A slight shift was observed in all three peaks, which was attributed to the reaction between the adsorbent and adsorbate during adsorption. The results of the relative contents of functional groups were listed in Table 2. After carotenoid adsorption, the molar percentage of C-P=O (relative to the total P) evidently decreased from 30 to 4.5%. In contrast, the molar percentage of C-P-O increased from 50 to 56.6%. This indicated that the C-P=O group served as an active component for carotenoid adsorption and transformed into the C-P-O group (35). Therefore, the enhanced functional groups and aromatic structure could facilitate the adsorption of carotenoids and improve the decolorization efficiency of camellia oil by CS-based modified activated carbon.

**TABLE 2:** Changes in chemical valence states changes of C, O, and P on CSHAC before and after carotenoid adsorption.

Samples	C1s (%)			O1s (%)		P2p (%)		
			
	C=C/C-C	C-O/C-P	C=O/O-C=O	C=O/P=O	C-O/C-P	C-P=O	C-P-O	C-O-P
CSHAC	49.03	40.00	10.97	26.5	73.5	30	50	20
CSHAC/carotenoids	85.6	8.0	6.4	53.17	46.83	4.5	56.6	38.9

**TABLE 3:** Amounts of free fatty acid and peroxide value in camellia oil samples bleached with 2% CSHAC.

Treatment	Free fatty acid (mg/g)	Peroxide value (g/100 g)
Refined camellia oil	0.92 ± 0.0018	0.32 ± 0.0034
Bleaching camellia oil	0.76 ± 0.0064	0.24 ± 0.0005

Values were expressed as means ± standard deviation.

### Adsorption performance

#### Effect of sorbent material

**FIGURE 5:**
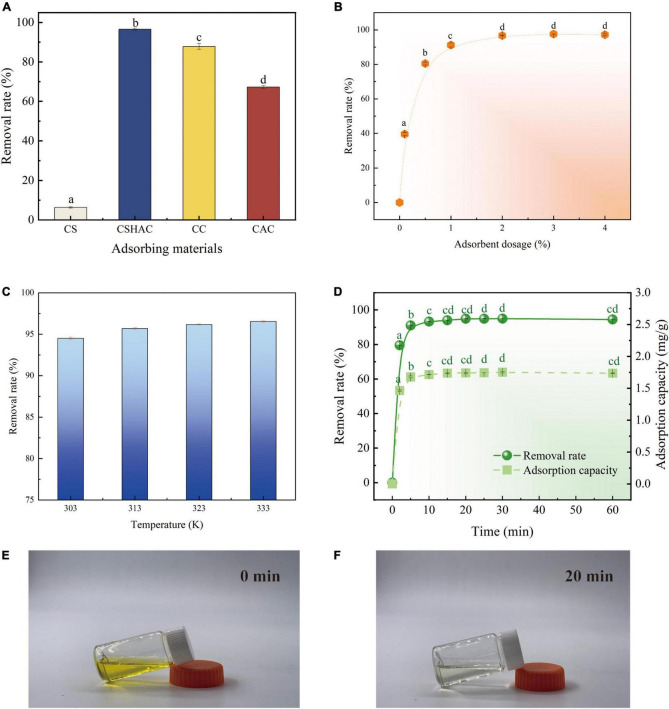
**(A)** Variations in carotenoid removal efficiency using various adsorbents and commercial decolorizers (adsorbent dosage is 2%; temperature is 333 K; time is 20 min). **(B)** Effect of CSHAC on carotenoid removal. **(C)** The carotenoid removal efficiency of CSHAC at adsorption equilibrium and different temperatures. **(D)** Contact time versus adsorption capacity and removal rate of CSHAC for carotenoids. **(E,F)** Photographs of refined camellia oil before and after adsorption for 20 min by CSHAC. Each value manifests the mean ± standard deviation of triplicate determinations and the mean values with diverse letters (a–d) differ significantly (*P* < 0.05).

By comparing CSH, CSHAC, CC, and CAC (Figure 5A), the carotenoid removal rate of CSHAC (96.5%) was the highest among the materials tested in the same conditions. The high specific surface area and three-dimensional pore structure of CSHAC exhibited high adsorption efficiency. The carotenoid removal efficiencies of commercial decolorization agents CC and CAC were 87 and 67%, respectively, highlighting the excellent adsorption effect of CSHAC. Hence, we concentrated on CSHAC and chose it as the best decolorization adsorbent for refined camellia oil.

#### Effect of CSHAC dosage

Figure 5B exhibited the effect of the CSHAC dosage on carotenoid adsorption performance. As the dosage of CSHAC increased from 0.01 to 2% (w/w), the carotenoid adsorption efficiency increased, reaching a plateau when the concentration of CSHAC exceeded 2%. The initial sharp increase was due to the augment in the specific surface area and abundant activation sites for carotenoid adsorption. The optimal CSHAC dose at which optimum carotenoid removal efficiency was achieved was 2%. Further experiments proceeded under these conditions. Moreover, it was evident from the data in Table 3 that peroxide and free fatty acids were removed using CSHAC. Therefore, the porous structure of the CS adsorbent might be effective for refining oil.

#### Effect of temperature

High carotenoid adsorption efficiency on CSHAC was achieved at high temperatures (Figure 5C). Adsorption was an endothermic process. The increase in temperature facilitated the oxidation and movement of carotenoid molecules in solution. The carotenoid removal efficiency increased when the temperature at 303–333 K.

The thermodynamic parameters were used for describing the thermodynamic behavior of carotenoid removal on CSHAC. Enthalpy (Δ*H*, kJ/mol), entropy [Δ*S*, kJ/(mol K)], and Gibbs free energy (Δ*G*, kJ/mol) were calculated as follows (37):


(4) $$K=\frac{q_{e}}{C_{e}}$$

(5) $$\ln K=\frac{-\Delta \,H}{R\,T}+\frac{\Delta \,S}{R}$$

(6) ΔG=−RTlnK

where *K* is a constant, *T* (K) is the absolute temperature, and *R* [8.314 J/(mol⋅K)] is the universal gas constant.

Table 4 showed the parameters of thermodynamics. A positive value of Δ*H* manifested the endothermic nature of the carotenoid adsorption (38). The value of Δ*S* was positive, which confirmed an increase in disorder at the solid-solution interface (39). The value of Δ*G* was negative at the four temperatures, which indicated that carotenoid adsorption onto CSHAC was spontaneous (40).

**TABLE 4:** Thermodynamic parameters for adsorption of carotenoids on CSHAC (CSHAC dosage, 2%).

Temperature (K)	Δ*H* (kJ/mol)	Δ*S* [J/(mol⋅K)]	Δ*G* (kJ/mol)
303	11.879875	39.40836	–3.506539
313			–535.9501
323			–879.929
333			–1193.325

#### Effect of contact time

An ultrafast adsorption rate was fulfilled on CSHAC, and the kinetic equilibrium could be accomplished rapidly (20 min) in refined camellia oil (Figure 5D). The ultrafast adsorption efficiency of CSHAC could be ascribed to three causes. (i) CSHAC (Figures 5E,F) manifested a morphology consisting of mesoporous, which facilitated the diffusion rate of adsorbate molecules. (ii) Abundant active groups (aromatic rings and oxygen functional groups) exposed on the surface of CSHAC could π–π conjugate with carotenoids, causing rapid adsorption. (iii) Hydrophobicity as one of the properties of CSHAC mainly contributed to an increase in the diffusion rate of carotenoids in the liquid membrane and pores of CSHAC, i.e., it greatly reduced the time for adsorption equilibrium.

### Adsorption kinetics

The kinetic behavior of carotenoid adsorption onto CSHAC was further simulated by using kinetic models: pseudo-first-order (PFO) kinetic and pseudo-second-order (PSO) kinetic models were given as Eqs 7 and 8, respectively. And the linear equation of intra-particle diffusion (IPD) was given by Eq. 9 (41). *q*_*e*_ and *q*_*t*_ are the adsorption capacities (mg/g) of CSHAC at equilibrium and time *t*, respectively; *k*_1_ (L/min), *k*_2_ (g/mg min), and *k*_*d*_ (mg/g min^0.5^) refer to rate constants; and *C* is a constant.


(7) $$\ln\left(q_{e}-q_{t}\right)=l\,n\,q_{e}-k_{1}\cdot t$$

(8) $$\frac{t}{q_{t}}=\frac{1}{k_{2}\cdot q_{e}^{2}}+\frac{t}{q_{e}}$$

(9) $$q_{t}=k_{d}\cdot t^{0.5}+C$$

**FIGURE 6:**
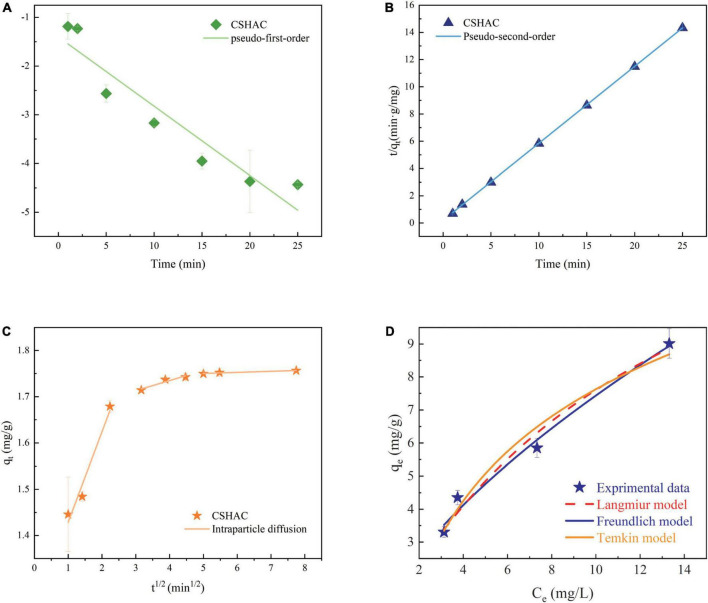
Kinetics model for adsorption of carotenoids. **(A)** PFO kinetics, **(B)** PSO kinetics, and **(C)** IPD. **(D)** Langmuir, Freundlich, and Temkin model descriptions of carotenoid adsorption onto CSHAC (CSHAC dosage, 2%; temperature, 333 K).

Figure 6 exhibited the experimental data fitted using the PFO, PSO, and IPD models. As is evident from the corresponding adsorption kinetics fitting parameters in Table 5, the PSO kinetics adsorption model exhibited a higher correlation coefficient value (*R*^2^) during the fitting of the carotenoid adsorption experimental data of CSHAC. Figure 6 also suggested that the theoretical adsorption capacity value fitted by the PSO model was closer to the experimental data of *q*_*e*_. Hence, carotenoid adsorption on CSHAC might be a chemical adsorption rate-controlling step (42). And the IPD model showed that the adsorption process had three stages, as shown in Figure 6. The first fitting stage indicated the spread of adsorbate molecules through the liquid film around the CSHAC and the outer adsorption on the sorbent surface. The second portion referred to the penetration of the adsorbate into the interlayer of the adsorbent. Finally, the plateau portion was ascribed to the equilibrium stage. In this work, the result of no plot passed the origin, manifesting that although the adsorption process contained IPD, it was not the only rate-limiting step (43). Therefore, carotenoid adsorption on CSHAC was affected in two manners: chemical adsorption and IPD.

**TABLE 5:** Kinetic models parameters for carotenoid adsorption on CSHAC.

PFO kinetic model			PSO kinetic model			IPD model					
*q*_*e,cal*_ (mg/g)	*k*_1_ (L/min)	R^2^	*q*_*e,cal*_ (mg/g)	*k*_2_ [g/(mg⋅min)]	R^2^	*k*_*d1*_ [mg/(g⋅min^0.5^)]	*R*_1_^2^	*k*_*d2*_ [mg/(g⋅min^0.5^)]	*R*_2_^2^	*k*_*d3*_ [mg/(g⋅min^0.5^)]	*R*_3_^2^
0.24	0.1381	0.9336	1.77	1.5803	0.9999	0.1952	0.9675	0.0218	0.9156	0.0023	0.9416

### Adsorption isotherms

The assessment of the adsorption isotherms is fundamental to depict the connection between the equilibrium adsorption capacity for carotenoids onto CSHAC and the remaining concentration of carotenoids at a fixed temperature. In order to understand the behavior of an adsorptive system, adsorption isotherm studies are necessary (32).

To simulate the monolayer adsorption behavior by using the Langmuir isotherm model (Eq. 10) (44):


(10) $$q_{e}=\frac{q_{m}\,k_{L}\,C_{e}}{1+k_{L}\,C_{e}}$$

where *q*_*e*_ (mg/g) and *q*_*m*_ (mg/g) are the equilibrium and maximum adsorption capacity of CSHAC for carotenoids, respectively. *k*_*L*_ (L/mg) is the adsorption constant of the Langmuir isotherm model.

To illustrate multilayer (≥ 2 layers) adsorption behavior by using the Freundlich isotherm model (Eq. 11) (45):


(11) $$q_{e}=k_{F}\,C_{e}^{\frac{1}{n}}$$

where *k*_*F*_ (mg/g) is the Freundlich model constant. *n* is a constant used for evaluating the intensity of the interaction between CSHAC and carotenoids.

The Temkin isotherm model suggests that the adsorption heat decreases linearly rather than logarithmically with the adsorption capacity. This model is appropriate for adsorption with a heterogeneous surface and strong interaction between the adsorbate and adsorbent. This model can be described by Eq. 12 (38):


(12) $$q_{e}=\frac{R\,T}{b_{T}}\,l\,n\,\left(a_{T}\,C_{e}\right)$$

where *a*_*T*_ (L/g) and *b*_*T*_ are the Temkin constants.

In order to evaluate the fitness of the models more comprehensively, the adjusted correlation coefficient (*adjR*^2^), chi-square (*χ^2^*), and residual sum of squares (*SSE*) were used to consider the influence of the number of parameters of various models by Eqs 13–15:


(13) $$adjR^{2}=1-\frac{\sum_{i=1}^{n}{\left(q_{e\,x\,p,i}-q_{c\,a\,l,i}\right)}^{2}}{\sum_{i=1}^{n}{\left(q_{e\,x\,p,i}-q_{m\,e\,a\,n}\right)}^{2}}\cdot \frac{\left(N-1\right)}{\left(N-P\right)}$$

(14) $$\chi \,2=\sum_{i=1}^{n}\frac{{\left(q_{e\,x\,p,i}-q_{c\,a\,l,i}\right)}^{2}}{q_{c\,a\,l,i}}$$

(15) $$S\,S\,E=\sum_{i=1}^{n}{\left(q_{e\,x\,p,i}-q_{c\,a\,l,i}\right)}^{2}$$

The adsorption isotherm experiment of CSHAC on adsorbing carotenoids with various concentrations of CSHAC (0.25, 0.5, 0.75, and 1%) at 333 K. As depicted in Figure 6D, Langmuir, Freundlich, and Temkin isotherm models were used to fit the experimental data. The corresponding parameters were listed in Table 6. The correlation coefficients, such as *adjR*^2^, *χ^2^*, and *SSE* values (Table 6) indicated that the adsorption of carotenoids onto CSHAC could be more accurately using the Freundlich model than the other models. Thus, the results manifested that the adsorption of carotenoids by CSHAC was a surface multilayer process (46). The result of 0 < 1/*n* < 1, after fitting using the Freundlich model, further indicated that carotenoids were easily adsorbed by CSHAC. For the Temkin model, the fitting results were reasonable, manifesting high correlation coefficients. It could be concluded that there were strong π–π electron-donor-acceptor interactions between CSHAC and carotenoid molecules (47).

**TABLE 6:** The adsorption isotherm model parameters of the Langmuir, Freundlich, and Temkin models for carotenoid adsorption on CSHAC (CSHAC dosage, 2%).

Model	Parameters	Evaluation			
		
		*R* ^2^	*AdjR* ^2^	χ^2^	*SSE*
Langmuir model	*q*_*m*_ = 17.63 mg/g; *k*_*L*_ = 0.08 L/mg	0.9762	0.9642	0.22	0.44
Freundlich model	*k*_*F*_ = 1.69 mg/g; *n* = 1.56	0.9853	0.9779	0.14	0.27
Temkin model	*a*_*T*_ = 0.79 L/mg; *b*_*T*_ = 751.38	0.9657	0.9485	0.32	0.64

### Proposed adsorption mechanism

**FIGURE 7:**
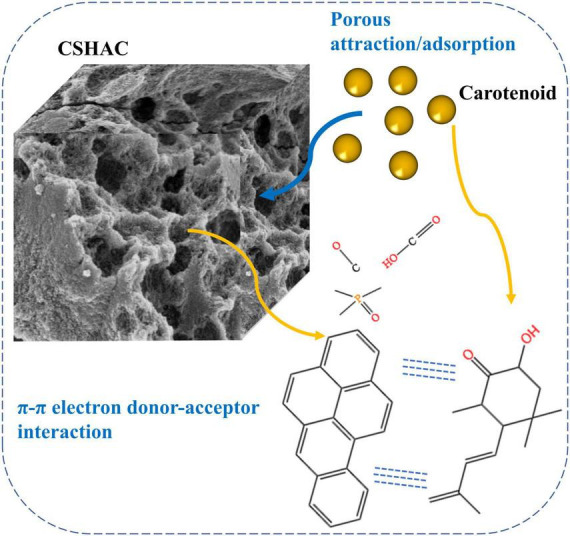
Proposed mechanisms for carotenoid adsorption on CSHAC.

Adsorption mechanisms (Figure 7) were proposed according to the structural characteristics of CSHAC and carotenoids depicted in available studies (48, 49) and the results reported herein. XPS results indicated that the presence of C=C, C-O, C-P=O, and O-C=O groups on the CSHAC surface was likely to facilitate the uptake of carotenoids. Owing to the structural characteristics of carotenoid molecules, they were prone to π–π interactions with CSHAC during adsorption. In addition, carotenoids were adsorbed onto the inner structure of IPD. The role of the inner structure was to pre-adsorb and increase the surface area and pore volume to provide more active sites. In summary, the superior adsorption performance of CSHAC was mainly ascribed to the number of C-, O-, and P-containing functional groups and its exceedingly high pore volume.

## Conclusion

In this study, p-doped activated carbon (CSHAC) was successfully produced using a simple method and used for efficient adsorption of carotenoids from refined camellia oil. The results showed that CSHAC had a better adsorption capacity for carotenoids than those of existing commercial decolorizers. This remarkable performance of CSHAC was ascribed to its stereoscopic structure with massive micropores and mesopores and the richness of active sites. The adsorption process of CSHAC was primarily dominated by chemical adsorption, with C=C, C-P=O, C=O, and O-C=O groups as active components, and the mechanism might be the π–π interactions between carotenoids and CSHAC. Pore filling facilitated the physical adsorption of carotenoids by CSHAC. Therefore, CSHAC has the potential to substitute commercial adsorbents for decolorization of camellia oil.

## Data availability statement

The original contributions presented in this study are included in the article/supplementary material, further inquiries can be directed to the corresponding author.

## Author contributions

RT, YL, DC, LG, and ND performed the experiments. RT analyzed the data and wrote the original manuscript. JY and DH revised the manuscript. HL, WL, and KL conceived and designed the experiments. All authors reviewed the manuscript and approved the submitted version.
